# Superiority of stapled side-to-side gastrojejunostomy over conventional hand-sewn end-to-side gastrojejunostomy for reducing the risk of primary delayed gastric emptying after subtotal stomach-preserving pancreaticoduodenectomy

**DOI:** 10.1007/s00595-017-1504-z

**Published:** 2017-03-23

**Authors:** Yasuhiro Murata, Akihiro Tanemura, Hiroyuki Kato, Naohisa Kuriyama, Yoshinori Azumi, Masashi Kishiwada, Shugo Mizuno, Masanobu Usui, Hiroyuki Sakurai, Shuji Isaji

**Affiliations:** Hepatobiliary Pancreatic and Transplant Surgery, Mie University School of Medicine, 2-174 Edobashi, Tsu, Mie 514-8507 Japan

**Keywords:** Subtotal stomach-preserving pancreaticoduodenectomy, Delayed gastric emptying, Stapled side-to-side anastomosis, Hand-sewn end-to-side anastomosis

## Abstract

**Background and purpose:**

Delayed gastric emptying (DGE) is the most common complication following pancreaticoduodenectomy (PD). The clinical efficacy of stapled side-to-side anastomosis using a laparoscopic stapling device during alimentary reconstruction in PD is not well understood and its superiority over conventional hand-sewn end-to-side anastomosis remains controversial. The objective of this study was to evaluate the effectiveness of the stapled side-to-side anastomosis in preventing the development of DGE after PD.

**Methods:**

The subjects of this retrospective study were 137 patients who underwent pancreaticoduodenectomy, as subtotal stomach-preserving pancreaticoduodenectomy (SSPPD; *n* = 130), or conventional whipple procedure (*n* = 7) with Child reconstruction, between January 2010 and May 2014. The patients were divided into two groups according to whether they had had a stapled side-to-side anastomosis (SA group; *n* = 57) or a conventional hand-sewn end-to-side anastomosis (HA group; *n* = 80).

**Results:**

SA reduced the operative time (SA vs. HA: 508 vs. 557 min, *p* = 0.028) and the incidence of delayed gastric emptying (SA vs. HA: 21.1 vs. 46.3%, *p* = 0.003) and was associated with shorter hospitalization (SA vs. HA: 33 vs. 39.5 days, *p* = 0.007). In this cohort, SA was the only significant factor contributing to a reduction in the incidence of DGE (*p* = 0.002).

**Conclusions:**

Stapled side-to-side gastrojejunostomy reduced the operative time and the incidence of DGE following PD with Child reconstruction, thereby also reducing the length of hospitalization.

## Introduction

Delayed gastric emptying (DGE) is the most common postoperative complication following pancreaticoduodenectomy (PD), with an incidence ranging from 18 to 59% [1–9]. Although it is not a life-threatening complication, it compromises quality of life and prolongs the hospital stay, thereby contributing to increased hospital costs. The cause of DGE remains unclear but seems to be multifactorial. Several surgical techniques have been correlated with DGE, including the type of PD, (classical Whipple procedure vs. pylorus-preserving PD; PPPD) [10], the type of reconstruction (Billroth I vs. Billroth II) [11], the method of reconstruction of gastric drainage (antecolic vs. retrocolic) [4], and the addition of Braun enteroenterostomy (Braun vs. no Braun) [12, 13]. However, the most effective technique for minimizing the incidence of DGE following PD is still being debated.

With advances in laparoscopic surgical techniques, a stapled anastomosis using a linear stapling device is now widely used during alimentary tract reconstruction. The Roux-en-Y anastomosis with a laparoscopic linear stapling device, where gastrojejunostomy is performed using a functional end-to-end anastomosis, is a common reconstruction technique after laparoscopic distal gastrectomy [14–16]. The advantages of the stapled side-to-side anastomosis include a standardized approach irrespective of the surgeon, relative ease as a reconstruction technique, a potential decrease in anastomotic leaks [17], and avoidance of anastomotic edema and subsequent stricture formation [18]. However, the clinical efficacy of stapled side-to-side anastomosis for reducing the risk of DGE, and its superiority over conventional hand-sewn end-to-side anastomosis following PD remain uncertain.

To our knowledge, there have been only two cohort studies examining the effectiveness of stapled side-to-side anastomosis for reducing the incidence of DGE after PD. A Japanese group found that DGE developed less frequently after stapled reconstruction than after hand-sewn reconstruction and that stapled reconstruction also reduced the length of the hospital stay [1, 19]. However, in their studies, a circular stapler was used primarily during duodenojejunostomy in 53 patients, whereas a linear stapler was used during gastrojejunostomy in only six patients with Roux-en-Y reconstruction [1]. A recent retrospective cohort study revealed that making a 9-cm long side-to-side gastrojejunostomy in the anterior surface of stomach, using a laparoscopic linear stapler was associated with a decreased incidence of DGE after standard PD in 84 patients vs. hand-sewn duodenojejunotomy after PPPD in 82 patients vs. stapled gastrojejunostomy on the distal anterior surface of the stomach with a 4 to 5 cm anastomotic length after standard PD in 28 patients [20].

In a recent study comparing the clinical outcomes of two different configurations of hand-sewn gastrojejunostomy, namely the side-to-side (*n* = 80) vs. end-to-side configuration (*n* = 80), the side-to-side configuration was associated with a reduced incidence of DGE after subtotal stomach-preserving PD (SSPPD) [21]. These observations led us to hypothesize that the stapled greater curvature side-to-side anastomosis using a laparoscopic linear stapler is the most effective procedure for preventing the development of DGE after SSPPD.

Since January 2010, we have been performing mainly SSPPD for the surgical treatment of pancreatic head and periampullary lesions. In September 2012, we introduced stapled side-to-side gastric posterior greater curvature-to-jejunal anastomosis using the 60 mm endoscopic linear stapler and stapled Braun jejunojejunostomy using the 45 mm endoscopic linear stapler, instead of conventional hand-sewn end-to-side gastric stump-to-jejunal anastomosis, to prevent the development of DGE following SSPPD. The objective of this study is to establish whether stapled side-to-side gastric posterior greater curvature-to-jejunal anastomosis is superior to conventional hand-sewn anastomosis for reducing the incidence of DGE after PD.

## Materials and methods

### Patient population

We reviewed the medical records of 137 consecutive patients who underwent PD at our institution between January 2010 and May 2014. The mean age was 68 years (37–85 years) and the patient population consisted of 80 men and 57 women. Primary diseases included pancreatic adenocarcinoma (*n* = 67), intraductal papillary mucinous neoplasm (IPMN) (*n* = 23), bile duct cancer (*n* = 14), ampullary cancer (*n* = 11), neuroendocrine tumor (*n* = 6), and various other diseases (*n* = 16). Among the 67 patients with pancreatic adenocarcinoma, 57 had received preoperative chemoradiotherapy (CRT) using a previously described protocol [22]. Eighty patients with obstructive jaundice underwent preoperative biliary drainage. Five senior attending surgeons (SI, MT, HS, MU, SM), with at least 15 years (15–34 years) experience, supervised all operations. The median experience of the operating surgeons was 14 years (7–34 years). This study protocol was approved by the medical ethics committee of Mie University School of Medicine (Approval number: 1524).

### Surgical procedures for PD

Surgical procedures included SSPPD (*n* = 130) and conventional PD (*n* = 7). Eleven of these patients underwent laparoscopy-assisted SSPPD. When dividing the gastrointestinal tract, the stomach was divided 2–3 cm proximal to the pyloric ring. In conventional PD, distal gastrectomy was performed as described previously [23]. The right gastroepiploic vessels, the right gastric artery, and the left gastric vein were routinely divided. For patients with malignancy, lymphadenectomy was performed, including dissection of the hepatoduodenal ligament, the common hepatic artery, portal vein, superior mesenteric vein, celiac trunk, and the superior mesenteric artery. For patients with pancreatic adenocarcinoma, the nerve plexus was dissected around the superior mesenteric artery (SMA) as described previously [24]. Other organs were resected for the portal vein/superior mesenteric vein in 63 patients, for the hepatic artery in five patients, for the splenic artery in eight patients, and for the colon in 12 patients.

After resection, surgical reconstruction was performed using a modification of the Child’s method. In patients who underwent laparoscopy-assisted SSPPD, reconstructions were performed through a 6-cm transverse incision. The proximal jejunal stump was passed through the antecolic pathway, and pancreaticojejunostomy, hepaticojejunostomy, and gastrojejunostomy were performed as described previously [25]. In 128 of the 137 patients, pancreaticojejunostomy was performed in an end-to-side fashion by duct-to-mucosa anastomosis using the pair-watch suturing technique [25]. The dunking method was used in seven patients, and other methods were used in two patients. A Braun anastomosis with side-to-side jejunojejunostomy was performed to prevent the back-flow of pancreatic and bile fluids into the stomach. An enteral feeding tube was placed in the jejunum from the afferent loop of Brawn anastomosis, using the Witzel technique. Two closed-system drains (J-VAC™ drainage system; Ethicon, Inc, Somerville, NJ, USA) were placed: one in the Winslow foramen and one around the pancreaticojejunostomy.

### Conventional hand-sewn end-to-side gastric stump-to-jejunal anastomosis and Braun anastomosis

**Fig. 1:**
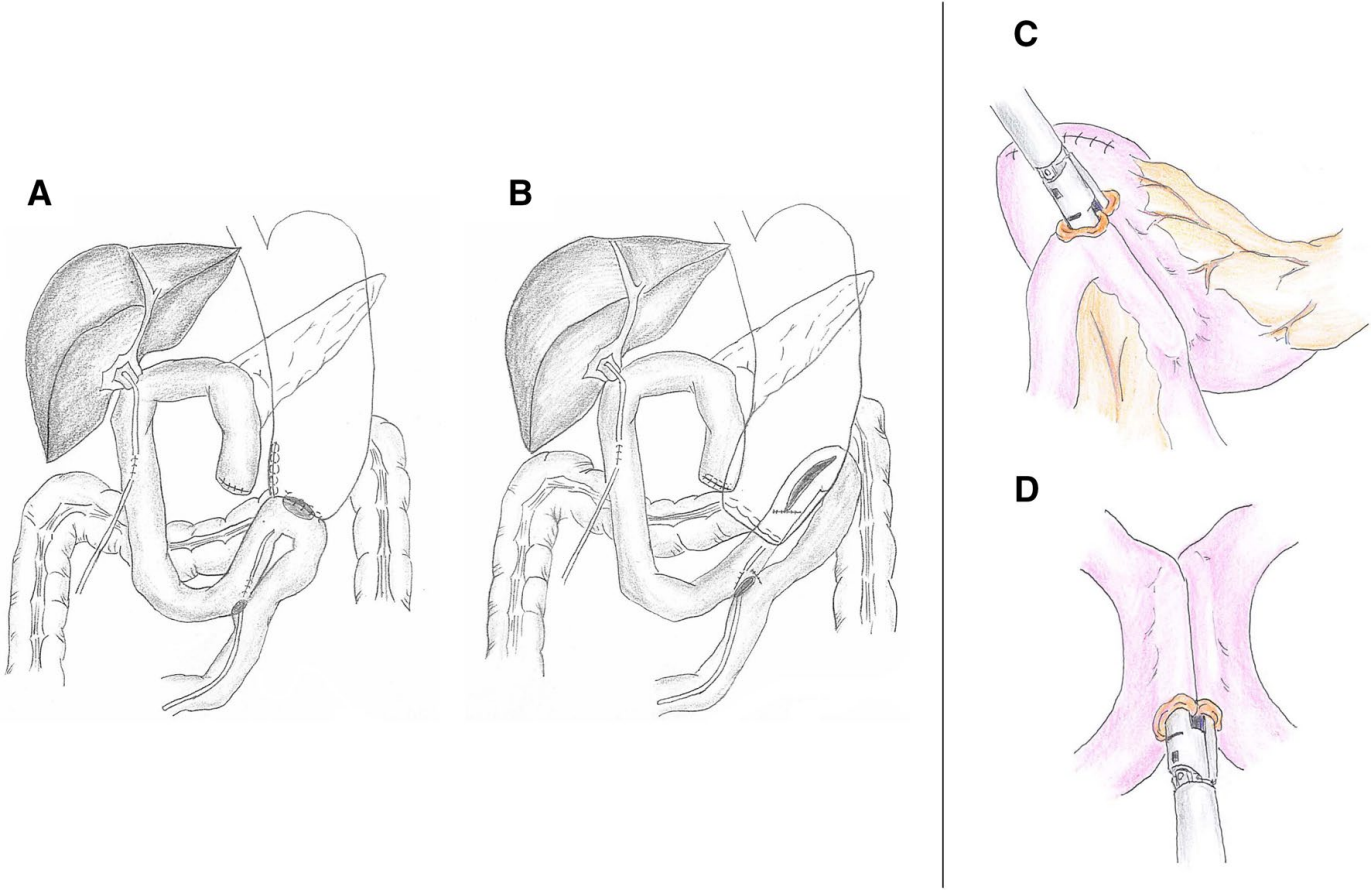
The procedure for stapled side-to-side anastomosis in gastrojejunostomy and Braun’s anastomosis using an endoscopic linear stapler. By September 2012, gastrojejunostomy and Braun jejunojejunostomy were being performed routinely in an end-to-side and side-to-side fashion, respectively, using an Albert-Lembert anastomosis (**a**). In September 2012, a stapled side-to-side anastomosis using a laparoscopic linear stapler was introduced for alimentary reconstruction during SSPPD (**b**). In terms of stapled side-to-side anastomosis, the gastrojejunostomy was performed with a side-to-side anastomosis in the posterior wall of the remaining stomach, approximately 3 cm from the cut end along the greater omentum, using a 60 mm endo-stapler (**c**). The Braun’s anastomosis was performed with a side-to-side anastomosis on the opposite side of the mesentery using a 45 mm endo-stapler (**d**)

By September 2012, we were performing gastrojejunostomy and Braun jejunojejunostomy routinely in an end-to-side and side-to-side fashion, respectively, using the Albert-Lembert anastomosis (Fig. 1a). Briefly, the greater curvature, approximately 5–6 cm from the gastric stump, was anastomosed to the jejunal loop in an end-to-side fashion. Full thickness approximation (Albert suturing) was started from the posterior wall, using a continuous pattern, followed by full thickness approximation of the anterior wall, using 4–0 absorbable sutures. Anterior and posterior seromuscular sutures were then placed using an interrupted pattern. Similarly, Braun’s anastomosis to a length of 3–4cm was made by side-to-side reconstruction in the opposite side of the mesentery.

### Stapled side-to-side gastric posterior greater curvature-to-jejunal anastomosis and stapled Braun anastomosis

In September 2012, we introduced stapled side-to-side anastomosis using a laparoscopic linear stapler for alimentary reconstruction during PD (Fig. 1b). The gastrojejunostomy and Braun jejunojejunostomy were conducted in a side-to-side fashion using the Endo GIA Tri-staple (Endo GIA™ 60 mm Articulating Medium/Thick Reload with Tri-Staple™ Technology, COVIDIEN Autosuture, Mansfield, MA, USA). An antecolic gastrojejunostomy was made via side-to-side reconstruction in the posterior wall of the remaining stomach, approximately 3 cm proximal to the distal staple line and 2 cm caudal to the greater omentum of the stomach (Fig. 1c). Similarly, Braun’s anastomosis was made by side-to-side reconstruction on the opposite side of the mesentery, using an endo-stapler (Endo GIA™ 45 mm Articulating Medium/Thick Reload with Tri-Staple™ Technology, COVIDIEN Autosuture, Mansfield, MA, USA) (Fig. 1d). The common entry hole was closed with a one-layer interrupted hand-sewn suture.

### Postoperative management

Both groups were managed according to the same clinical pathway. The nasogastric (NG) tube was removed when the amount of postoperative drainage was below 500 ml/day. Oral intake of fluids was recommenced routinely on postoperative day (POD) 3 and solids were introduced over the following days. Erythromycin or octreotide were not given perioperatively. The nasogastric tube was reinserted if the patient had nausea or vomiting and/or if severe distention of the stomach was observed on abdominal radiography. Serum amylase and abdominal drain fluid amylase were measured on or after POD 3. The abdominal drain was removed on POD 5 to 6, if there were no signs of pancreatic fistula or intra-abdominal collections.

### Definition of outcome measures

Patients were divided into two groups according to the method of alimentary reconstruction used: a stapled side-to-side anastomosis group (group SA; *n* = 57) and a conventional hand-sewn end-to-side anastomosis group (group HA; *n* = 80). We compared patient characteristics, surgical parameters, the intraoperative factors of operative time and blood loss, and the following surgical outcomes: the amount of NG tube discharge on PODs 1 and 3, the duration of NG tube placement, the incidence of recurrent gastric drainage, the number of days until a liquid diet was recommenced, the incidence of delayed gastric emptying (DGE), the frequency of ingestion of solid food on POD 14, the incidence of postoperative pancreatic fistula (POPF), and the length of hospitalization. The incidence of DGE was calculated according to the International Study Group of Pancreatic Surgery’s (ISGPS) web-based calculator (http://pancreasclub.com/calculators/isgps-calculator/) [9]. Postoperative pancreatic fistula was defined as a drain fluid amylase concentration more than three times greater than the upper range of serum amylase concentration on POD 3, according to the International Study Group on Pancreatic Fistula [26]. Postoperative pancreatic fistula was classified into grade A, B, or C, according to severity. Grade A fistulas were “transient fistulas” not associated with a delay in hospital discharge. Grade B fistulas led to a delay in discharge, with persistent drainage for more than 3 weeks. Grade C fistulas were usually associated with major complications. Grades B and C DGE and POPF were defined as clinically relevant surgical complications.

Because postoperative intra-abdominal complications are major causes of DGE [2, 5, 6, 27], we defined DGE associated with any postoperative intra-abdominal complications as secondary DGE, whereas DGE without any intra-abdominal complications was defined as primary DGE. According to the ISGPF definition, obstruction of the gastrojejunostomy from a technical problem at the anastomosis such as an anastomotic stricture or a small bowel obstruction close to the gastrojejunostomy were not classified as DGE [26]. In the present study, we did not evaluate the incidence of obstruction of the gastrojejunostomy caused by a technical problem at the anastomosis on imaging studies. However, we considered that there were no cases of technical problems with anastomosis because all patients with DGE could be managed without any specific treatment for the anastomosis such as bougie and re-anastomosis. As for postoperative intra-abdominal complications associated with secondary DGE, they included POPF, anastomotic leakage, intra-abdominal abscess, fluid collection or bleeding, surgical site infection, ileus, gastrointestinal infection or bleeding, thrombus of the portal vein/superior mesenteric vein, and liver infarction. These complications, apart from POPF and DGE, were classified according to the criteria proposed by Clavien and Dindo [28]. Grade II or more complications were recorded.

### Analysis of risk factors for DGE

Risk factors for DGE were analyzed in a univariate analysis and included age, sex, body mass index, prevalence of diabetes mellitus, preoperative biliary drainage, disease (pancreatic adenocarcinoma vs. others), preoperative chemoradiation therapy, surgical procedures (SSPPD vs. conventional PD), laparoscopic-assisted PD, portal vein resection, experience of the surgeons (15 years or more vs. less than 15 years), operative time (min), blood loss (mL), method of alimentary reconstruction (stapled side-to-side anastomosis vs. hand-sewn end-to-side anastomosis), POPF (absent, grade A, B, or C), and intra-abdominal complications (yes or no). A logistic regression model was used to determine independent risk factors for postoperative DGE as well as primary DGE. The independent risk factors of the variables are expressed as odds ratios (OR) with their 95% confidence intervals (CI).

### Statistical analysis

We compared patient characteristics, surgical parameters, and surgical outcomes between the patients in the SA and HA groups and analyzed the risk factors for DGE. Continuous variables are expressed as the median (range) and compared using the Wilcoxon test. Categorical variables were compared using either the Chi-square test or Fisher’s exact test. All statistical analyses were performed with JMP® Pro 9.0.2 (SAS Institute Inc., Cary, NC). *P* values less than 0.05 were considered significant.

## Results

### Surgical outcome of PD

For the 137 patients who underwent PD, the median intraoperative blood loss was 843 mL (5–11937 mL), the median operative time was 542 min (327–832 min), and the median hospital stay was 36 days (16–103 days). The median amount of NG tube discharge was 105 mL (0–1290 mL, *n* = 137) on POD 1 and 140 mL (0–1730 mL, *n* = 59) on POD 3 and the median duration of NG tube placement was 2 days (1–45 days). All 137 patients resumed a liquid diet on POD 7 (3–49 days) and 136 resumed a solid diet on POD 9 (4–77 days).

Table 1 summarizes the surgical complications. The overall PD complication rate was 55.5%. DGE developed in 49/137 patients (35.8%), with an incidence of clinically relevant grade B or C of 19.7%. Twenty-one of the 49 patients with DGE reported postoperative intra-abdominal complications (42.9%, secondary DGE). In 28 patients, DGE was not associated with postoperative intra-abdominal complications (57.1%, primary DGE). The rate of non-DGE complications was 46.0%, and the rate of intra-abdominal complications other than DGE was 39.4%. Eighteen patients (13.1%) suffered grade B or C POPF. The 30-day postoperative and in-hospital mortality rates were 0.7% (*n* = 1) and 1.5% (*n* = 2), respectively. There were two hospital deaths directly related to surgery. One patient who underwent SSPPD with combined resection of the common hepatic artery and portal vein for pancreatic adenocarcinoma died secondary to sepsis after grade C POPF and subsequent anastomotic leakage of the gastrojejunostomy. The other patient died secondary to sepsis from serious postoperative pneumonia.

**Table 1:** Postoperative complications after pancreaticoduodenectomy (*n* = 137)

Complication	Grade	*N*	(%)
DGE (*n* = 49, 35.8%)	A	22	16.1
	B	17	12.4
	C	10	7.3
POPF (*n* = 21, 15.3%)*	A	3	2.2
	B	15	10.9
	C	3	2.2
Acute cholangitis (*n* = 6, 4.4%)*	II	6	4.4
Surgical site infection (*n* = 5, 3.6%)*	II	1	0.7
	IIIa	3	2.2
	IIIb	1	0.7
Biliary fistula (*n* = 4, 2.9%)*	IIIa	3	2.2
	IIIb	1	0.7
Intra-abdominal abscess (*n* = 4, 2.9%)*	IIIa	4	2.9
Intra-abdominal fluid collection (*n* = 4, 2.9%)*	II	2	1.5
	IIIa	2	1.5
Ileus (*n* = 4, 2.9%)*	IIIa	1	0.7
	IIIb	3	2.2
Gastrointestinal bleeding (*n* = 4, 2.9%)*	IIIa	3	2.2
	IVa	1	0.7
Enterocolitis (*n* = 4, 2.9%)*	II	4	2.9
Liver infarction (*n* = 3, 2.2%)*	II	3	2.2
Intra-abdominal bleeding (*n* = 3, 2.2%)*	IIIb	1	0.7
	IVa	2	1.5
Others (*n* = 10, 7.3%)			
Cerebral infarction	II	1	0.7
Sepsis caused by MRSA infection	II	1	0.7
Thrombus of SMV/PV*	II	1	0.7
Psudoaneurysm of splenic artery*	IIIa	2	1.5
Psudoaneurysm of hepatic artery*	IIIa	1	0.7
Intractable ascites*	IIIa	1	0.7
Leakage of colon anastomosis*	IIIb	1	0.7
Cardiac failure	IVa	1	0.7
Aspiration pneumonia	V	1	0.7

*Intra-abdominal complications

*DGE* delayed gastric emptying, *POPF* postoperative pancreatic fistula, *MRSA* Methicillin-resistant *Staphylococcus aureus, SMV*/*PV* superior mesenteric vein/ portal vein

Regarding complications related to reconstruction of the alimentary tract, one patient (1.3%) who underwent hand-sewn anastomosis suffered anastomotic leakage of the gastrojejunostomy after POPF. No anastomotic leakage was found in the group with stapled side-to-side anastomosis. One patient (1.3%) who underwent hand-sewn anastomosis suffered postoperative anastomotic bleeding from the gastrojejunostomy, while three patients (5.3%) who underwent stapled anastomosis suffered postoperative anastomotic bleeding from the gastrojejunostomy (*n* = 2) and Braun jejunojejunostomy (*n* = 1). There was no significant difference in the incidence of postoperative anastomotic bleeding. Endoscopic hemostasis was carried out successfully for the anastomotic bleeding in all these patients.

### Patient-related factors

Table 2 shows the patient characteristics and surgical parameters in group SA vs. group HA. Preoperative factors, including age, sex, prevalence of diabetes mellitus, performance of preoperative biliary drainage, disease (pancreatic adenocarcinoma vs. others), and performance of preoperative chemoradiation therapy were comparable between the groups. The surgical procedures (SSPPD vs. conventional PD), surgical approach (open vs. laparoscopic surgery), and frequency of portal vein resection (not performed vs. performed) were similar in the two groups. The experience level of the surgeons was not significantly different.

**Table 2:** Clinical characteristics of the patients in the side-to-side anastomosis vs. the hand-sewn anastomosis groups

		Group SA (*n* = 57)	Group HA (*n* = 80)	*P* value
Patient characteristics				
Age		68 (37–82)	67 (39–85)	0.95
Gender	Male/female	30/27	50/30	0.25
Body mass index		20.7 (15.1–33.4)	21.4 (14.1–38.8)	0.06
Diabetes mellitus	Yes/no	13/44	18/62	0.97
Preoperative biliary drainage	Yes/no	34/23	46/34	0.8
Pancreatic adenocarcinoma	Yes/no	28/29	39/41	0.97
Preoperative chemoradiation	Yes/no	27/30	30/50	0.25
Surgical parameters				
Operative procedure	SSPPD	55	75	0.46
	Conventional PD	2	5	
Laparoscopic-assisted PD	Yes/no	7/50	4/76	0.13
Combined resection of portal vein	Yes/no	28/29	35/45	0.53
Year of surgeon experience>/=15 years	Yes/no	23/34	32/48	0.97

*SA* stapled side-to-side anastomosis, *HA* conventional hand-sewn end-to-side anastomosis, *SSPPD* subtotal stomach preserved pancreatoduodenectomy, *PD* pancreatoduodenectomy

### Surgical outcome and the occurrence of DGE

Table 3 shows the surgical outcome of PD according to the method of alimentary reconstruction. The operative time was significantly shorter in the SA group than in the HA group (508 vs. 557 min, respectively; *p* = 0.028). The amount of NG tube drainage on POD 1 was significantly less in the SA group than in the HA group (50 vs. 165 mL, respectively; *p* = 0.0001). The duration of NG tube placement was significantly shorter in the SA group than in the HA group (1 vs. 3 days, respectively; *p* < 0.0001). The number of days until a liquid diet was initiated was also significantly less in the SA group (5 vs. 7 days, respectively; *p* < 0.0001). The overall incidence of DGE was significantly lower in the SA group than in the HA group (21.1 vs. 46.3%, respectively; *p* = 0.003). Based on the mechanism of DGE, the incidence of primary DGE was significantly lower in the SA group than in the HA group (8.8 vs. 28.8%, respectively; *p* = 0.002). In contrast, the incidence of secondary DGE was comparable between the groups, suggesting that stapled side-to-side anastomosis prevents primary DGE more efficiently than hand-sewn anastomosis, but does not affect the development of secondary DGE. Clinically relevant grade B or C DGE was significantly less frequent in the SA group than in the HA group (7.0 vs. 28.8%, respectively; *p* = 0.005). The incidence of grade B DGE alone was significantly lower in the SA group (3.5 vs. 18.8%, respectively; *p* = 0.008), while those of grade A and grade C DGE did not differ significantly between the groups. The rate of ingestion of solid food on POD 14 was significantly higher in the SA group than in the HA group (68.4 vs. 48.8%, respectively; *p* = 0.021).

**Table 3:** Surgical outcome after pancreaticoduodenectomy in the side-to-side anastomosis vs. the hand-sewn anastomosis groups

	Group SA (*n* = 57)	Group HA (*n* = 80)	*P* value
Operative time (min)	508 (330–818)	557 (327–832)	0.028*
Blood loss (ml)	820 (5–11937)	853 (150–2880)	0.24
Amount of NG tube drainage			
POD1 (ml)	50 (0–1050)	165 (0–1290)	0.0001*
POD3 (ml)	100 (0–790)	200 (0–1730)	0.39
Duration of NG tube placement (days)	1 (1–45)	3 (1–27)	<0.0001*
Re-insertion of NG tube	4 (7.0%)	13 (16.3%)	0.096
Days until initiation of liquid diet (days)	5 (3–49)	7 (3–34)	<0.0001*
Incidence of DGE			
Overall incidence	12 (21.1%)	37 (46.3%)	0.003*
Primary DGE	5 (8.8%)	23 (28.8%)	0.002*
Secondary DGE	7 (12.3%)	14 (17.5%)	0.4
Grade of DGE			
Grade A	8 (14.0%)	14 (17.5%)	0.58
Grade B	2 (3.5%)	15 (18.8%)	0.008*
Grade C	2 (3.5%)	8 (10.0%)	0.194
Ingestion of solid food on POD14	39 (68.4%)	39 (48.8%)	0.021*
POPF (grade B or C)	8 (14.0%)	10 (12.5%)	0.79
Biliary fistula	1 (1.8%)	3 (3.8%)	0.64
Intra-abdominal abscess	3 (5.3%)	1 (1.3%)	0.31
Intra-abdominal fluid collection	4 (7.0%)	0 (0%)	0.028*
Ileus	2 (3.5%)	2 (2.5%)	1
Gastrointestinal bleeding	3 (5.3%)	1 (1.3%)	0.31
Intra-abdominal bleeding	2 (3.5%)	1 (1.3%)	0.57
Overall incidence of complications (C–D II or more)	34 (59.7%)	42 (52.5%)	0.41
Length of hospital stay (days)	33.0 (16–79)	39.5 (20–103)	0.0072*

* stastically significant p values (*p* < *0.05*)

*SSPPD* subtotal stomach preserved pancreatoduodenectomy, *NG* nasogastric tube, *POD* postoperative day, *Re-gastric drainage reinsertion* of nasogastric tube, *DGE* delayed gastric emptying, *POPF* postoperative pancreatic fistula graded according to the definition proposed by an international study group on pancreatic fistula (ISGPF), Intra-abdominal complications; postoperative abdominal complications except for DGE, *C-D* Clavien and Dindo grading, *SA* stapled side-to-side anastomosis, *HA* conventional hand-sewn end-to-side anastomosis

The incidences of grade B or C POPF and intra-abdominal abscess were not significantly different between the SA and HA groups (14.0 and 5.3 vs. 12.5 and 1.3%, respectively; *p* = 0.79, 0.31). The rates of intra-abdominal fluid collections were significantly higher in the SA group than in the HA group, but the overall incidence of grade II or more postoperative complications were similar. The length of hospital stay was significantly shorter in the SA group than in the HA group, at 33.0 vs. 39.5 days, respectively (*p* = 0.0072).

Taken together, these data indicate that stapled side-to-side anastomosis can reduce operative time, the incidence of DGE, and hospital stay remarkably, compared with hand-sewn end-to-side anastomosis.

### Demographic and comorbidity variables of patients with or without DGE

Table 4 compares the demographic and comorbidity variables between patients with vs. those without DGE. The method of alimentary reconstruction (stapled side-to-side anastomosis vs. hand-sewn end-to-side anastomosis) was the only significant risk factor for DGE (*p* = 0.002). In multivariate analysis, stapled side-to-side anastomosis was identified as an independent significant negative risk factor for DGE (OR, 95% CI: 0.269, 0.096–0.69, *p* = 0.006). Taken together, these data show that stapled side-to-side anastomosis reduced the incidence of DGE independently, irrespective of other factors. To clarify the efficacy of stapled side-to-side anastomosis for preventing primary DGE, the clinicopathological factors were similarly compared between patients with (*n* = 28) and those without DGE (*n* = 50) in the absence of intra-abdominal complications. Stapled side-to-side anastomosis was the only significant factor that reduced the incidence of primary DGE, suggesting that stapled side-to-side anastomosis is superior to conventional hand-sewn anastomosis for preventing primary DGE (*p* = 0.044, Table 5). In multivariate analysis, stapled side-to-side anastomosis was identified as a significant independent negative risk factor for primary DGE (OR, 95% CI: 0.224, 0.043–0.882, *p* = 0.032).

**Table 4:** Clinicopathological factors of patients with vs. those without delayed gastric emptying (*n* = 137)

		Without DGE (*n* = 88)	With DGE (*n* = 49)	Univariate *P* value	Multivariate *P* value (OR, 95% CI)
Patient characteristics					
Age		67.5 (37–85)	68 (41–82)	0.7	
Gender	Male/female	53/35	27/22	0.56	
Body mass index		20.9 (15.1–38.8)	21.5 (14.1–30.5)	0.93	
Diabetes mellitus	Yes/no	24/64	7/42	0.073	
Preoperative biliary drainage	Yes/no	51/37	29/20	0.89	
Pancreatic adenocarcinoma	Yes/no	45/43	22/27	0.48	
Preoperative chemoradiation	Yes/no	41/47	16/33	0.11	
Surgical parameters					
Surgical procedure	SSPPD/conventional PD	84/4	46/3	0.69	
Laparoscopic-assisted PD	Yes/no	7/81	4/45	0.97	
Portal vein resection	Yes/no	43/45	20/29	0.36	
Year of surgeon experience>/=15 years	Yes/no	37/51	18/31	0.54	
Operative time (min)		532.5 (330–818)	552 (327–832)	0.78	
Blood loss (ml)		848 (5–11937)	834 (50–6680)	0.34	
Method of alimentary reconstruction	HA/SA	43/45	37/12	0.002*	0.006* ( 0.269, 0.096–0.692 )
Postoperative factors					
POPF	Absent or grade A/grade B or C	78/10	41/8	0.42	
Intra-abdominal complications	Yes/no	35/53	19/30	0.91	

* stastically significant p values (*p* < *0.05*)

*DGE* delayed gastric emptying, *SSPPD* subtotal stomach preserved pancreatoduodenectomy, *PD* pancreatoduodenectomy, *HA* hand-sewn end to side anastomosis, *SA* stapled side-to-side anastomosis, *POPF* postoperative pancreatic fistula graded according to the definition proposed by an international study group on pancreatic fistula (ISGPF)

**Table 5:** Clinicopathological factors of patients with vs. those without primary delayed gastric emptying (*n* = 78)

		Without primary DGE (*n* = 50)	With primary DGE (*n* = 28)	Univariate *P* value	Multivariate *P* value (OR, 95% CI)
Patient characteristics					
Age		68 (37–85)	67.5 (44–81)	0.64	
Gender	Male/female	26/24	14/14	0.87	
Body mass index		20.9 (15.1–38.8)	20.9 (15.3–30.5)	0.49	
Diabetes mellitus	Yes/no	13/37	4/24	0.23	
Preoperative biliary drainage	Yes/no	28/22	16/12	0.92	
Pancreatic adenocarcinoma	Yes/no	27/23	13/15	0.52	
Preoperative chemoradiation	Yes/no	25/25	10/18	0.22	
Surgical parameters					
Surgical procedure	SSPPD/conventional PD	46/4	27/1	0.65	
Laparoscopic-assisted PD	Yes/no	2/48	1/27	1	
Portal vein resection	Yes/no	26/24	14/14	0.87	
Year of surgeon experience>/=15 years	Yes/no	19/31	11/17	0.91	
Operative time (min)		530 (330–800)	520 (327–813)	0.78	
Blood loss (ml)		800 (5–5089)	807 (200–6680)	0.93	
Method of alimentary reconstruction	HA/SA	30/20	23/5	0.044*	0.032* ( 0.224, 0.043–0.882 )

* stastically significant p values (*p* < *0.05 *)

*DGE* delayed gastric emptying, *SSPPD* subtotal stomach preserved pancreatoduodenectomy, *PD* pancreatoduodenectomy, *HA* hand-sewn end to side anastomosis, *SA* stapled side-to-side anastomosis

## Discussion

In the present study, DGE developed less frequently after stapled side-to-side anastomosis using a laparoscopic linear stapling device than after conventional hand-sewn reconstruction during gastrojejunostomy and Braun jejunojejunostomy in PD with Child reconstruction. This contributed to a shorter hospitalization. Moreover, stapled side-to-side anastomosis was an independent negative risk factor for the overall incidence of DGE and for primary DGE, but not for secondary DGE. These results suggest that our newly-introduced procedure of stapled side-to-side anastomosis can reduce the incidence of primary DGE better than conventional end-to-side hand-sewn anastomosis.

Advances in both surgical techniques and perioperative management have contributed to a decreased mortality of less than 2% after PD at high-volume centers [8, 29, 30]. However, the morbidity of PD remains high, with an overall complication rate of 40% [31]. DGE is the leading complication, but the reported incidence varies widely because there is no standard definition. Because of the variations in definition, the true morbidity of DGE has been difficult to assess. The ISGPS recently proposed a grading system to categorize the severity of DGE [26]. In the present study, DGE was diagnosed and graded precisely, using the ISGPS web-based calculator. This calculator uses the new standardized definition for POPF and includes a calculator for delayed gastric emptying (DGE) [9]. After clinical testing with actual data, the calculator uses a slightly modified definition from that published by the ISGPS. In the present study, the overall rate of DGE was 35.8%, and the combined rate of grades B and C DGE was 19.7% (grade B: 12.4%, grade C: 7.3%). In a past study that analyzed the rate of DGE using the ISGPS classification [2], the overall rate of DGE was 33.3%, and the combined rate of grades B and C DGE was 21.0% This is comparable to our results. The web-based calculator based on the ISGPS classification is a useful tool to precisely classify the grade of DGE based on universal criteria.

DGE can be initiated by anastomotic edema or stenosis following a disturbance in the blood supply, which may cause the progression of gastroparesis [32]. We consider that there are three main reasons for the stapled side-to-side anastomosis using a laparoscopic stapling device reducing the incidence of DGE vs. hand-sewn end-to-side anastomosis. First, the stapled side-to-side anastomosis can hold the anastomotic lumen open more uniformly and prevent anastomotic edema or stenosis more efficiently than the end-to-side hand-sewn anastomosis, even in the early postoperative period. Because the length of the end-to-side and side-to-side anastomoses was almost the same in the current study, the stapled anastomosis may cause stricture of the anastomosis less frequently than the hand-sewn anastomosis. Second, side-to-side gastrojejunostomy avoids disturbance of the blood supply to the anastomotic site, whereas the end-to-side hand-sewn gastrojejunostomy might be affected by local ischemia of the cut end of antrum. Third, the position of the anastomosis proximal to the gastric staple line, near the greater curvature, allows for the easier drainage of food contents into the jejunum. Two recent cohort studies compared the clinical outcomes of two different configurations of gastrojejunostomy after PD: the side-to-side vs. end-to-side configuration with either stapled or hand-sewn anastomosis [20, 21]. Both concluded that side-to-side configuration along the great curvature was superior to the antrum end-to-side fashion for reducing the incidence of DGE following PD. Therefore, the side-to-side configuration along the greater curvature might have contributed to the reduction in the incidence of DGE after PD in the current study.

The Braun jejunojejunostomy is thought to be an important procedure for reducing the risk of DGE [12, 33]. Because the Braun jejunojejunostomy can reduce the pressure in the biliopancreatic limb, it may decrease stimulation to the pressure receptors of the gastrointestinal tract, thereby decreasing the incidence of DGE. In our institution, we routinely add Braun jejunojejunostomy and have also modified the procedure. The jejunojejunostomy using a 45 mm linear stapler is able to obtain a wider anastomotic entry without causing edema of the anastomotic site. We speculate that these factors were associated with reducing the pressure in the gastrointestinal tract and the incidence of DGE.

The major disadvantage of stapled anastomosis is postoperative bleeding from the anastomotic site [1]. In our series, three patients (5.3%) who underwent stapled anastomosis suffered postoperative anastomotic bleeding from the gastrojejunostomy (*n* = 2) and Braun jejunojejunostomy (*n* = 1), whereas only one patient (1.3%) who underwent hand-sewn anastomosis had anastomotic bleeding from the gastrojejunostomy. This difference was not significant within our sample size. It will be necessary to evaluate the risk of bleeding from the stapled anastomotic site to establish the feasibility of stapled anastomosis during alimentary reconstruction after PD. Intraoperatively, it is important to visualize the stapled line carefully from the common entry hole and to achieve precise hemostasis if bleeding is identified. On the other hand, it has been reported that the stapled side-to-side anastomosis is beneficial for reducing the anastomotic leak rate compared with hand-sewn anastomosis [17]. In the present study, we did not encounter any anastomotic leakage in the patients who underwent stapled side-to-side anastomosis, whereas one patient who underwent hand-sewn anastomosis suffered anastomotic leakage from the gastrojejunostomy, caused by a postoperative pancreatic fistula. Further case evaluation will be needed to evaluate the benefit–risk ratio for the usage of a linear stapling device during PD.

The causes for DGE remain unclear and are probably multifactorial [34]. The possible factors associated with DGE include antroduodenal ischemia [35], postoperative intra-abdominal complications [1, 5, 6, 27, 36, 37], low plasma motilin concentration [3, 38], and technical factors such as torsion or angulation of the digestive tract reconstruction [32, 33, 39]. Among the several possible factors of DGE, the most important are postoperative intra-abdominal complications like pancreatic fistula, biliary fistula, and intra-abdominal abscess [35, 36]. Among these postoperative intra-abdominal complications, POPF is most significantly associated with an increased risk of DGE. Several retrospective studies report that POPF is an independent risk factor for DGE [1, 2, 36]. In the original study analyzing the incidence of DGE following PD in consecutive patients, using the ISGPS web-based calculator [9], the occurrence of clinically relevant DGE (grade B/C) was strongly associated with the presence of pancreatic anastomotic failure. Given this information, the present study retrospectively evaluated the potential risk factors for DGE, including intra-abdominal complications such as POPF. Unexpectedly, the incidence of POPF was not significantly different between the patients with vs. those without DGE (16.3 vs. 11.4%, *p* = 0.42). Similarly, the overall rate of postoperative intra-abdominal complications was not significantly different between patients with vs. those without DGE (39.8 vs. 38.8%, *p* = 0.91). On the other hand, stapled side-to-side anastomosis was the only independent negative risk factor for the overall incidence of DGE (Table 4). Furthermore, stapled side-to-side anastomosis was also the only significant negative risk factor for primary DGE (Table 5), but not associated with the development of secondary DGE. These data show that stapled side-to-side anastomosis reduces the risk of primary DGE, irrespective of the overall prevalence of possible causes of DGE, like postoperative intra-abdominal complications.

This study has several limitations. First it was a non-randomized retrospective study at a single institution and the procedures were performed during two separate time periods. Second, the comparison between the two types of anastomosis was influenced not only by the different techniques (stapled vs. hand-sewn), but also by the different sites and configurations of the anastomoses (posterior stomach wall side-to-side vs. antrum end-to-side) in the two groups. Therefore, it is unclear which site and technique (posterior stomach wall side-to-side vs. antrum end-to-side) or if using the stapling device (the use of staples vs. hand-sewn) had the greatest influence on reducing the incidence of gastric emptying. One of the major characteristics of the current study was the longer postoperative hospitalization period than that reported in studies from Western countries, most likely attributable to differences in medical insurance systems [31]. A prospective randomized trial should be planned to establish whether stapled side-to-side anastomosis is the best anastomotic technique for reducing the incidence of DGE, which would contribute to shortening the post-PD hospital length of stay.

## Conclusion

Stapled side-to-side anastomosis effectively reduced the incidence of DGE after PD with Child reconstruction, which consequently decreased the length of hospitalization.
